# Structure first – exploration and discovery with cryo-electron microscopy

**DOI:** 10.1242/jcs.264215

**Published:** 2026-02-26

**Authors:** Miguel Ricardo Leung

**Affiliations:** Hubrecht Institute-KNAW and University Medical Center Utrecht, Uppsalalaan 8, 3584 CT Utrecht, The Netherlands

**Keywords:** Cryo-electron microscopy, Cryo-electron tomography, *De novo* protein identification

## Abstract

The ability to directly observe living systems at finer levels of detail is a strong catalyst for biological discovery. This Perspective highlights how cryo-electron microscopy (cryo-EM) is enabling a ‘structure-first approach’ that can be harnessed for exploration and discovery at the molecular scale, as exemplified in recent studies across the diverse biological contexts curated here. Improvements in throughput, robustness and accessibility of cryo-EM have expanded the range of samples amenable to high-resolution structural analysis to include native protein complexes directly isolated from primary material or imaged unperturbed within the cellular environment. It is therefore increasingly common to encounter unknown proteins in cryo-EM studies, either as unexpected components of a known complex or as completely uncharacterized structures. Advancements in machine learning-assisted model building and protein structure prediction, aided by proteomics and cross-linking mass spectrometry, facilitate protein identification from cryo-EM maps over a wide resolution range, making it possible to derive molecular identity without any prior knowledge or need for specific labelling. In summary, cryo-EM has extended the reach of structural biology beyond focused structure determination of known targets to the exciting frontier of uncovering altogether new proteins and interactions.

## Teaser

**Summary:** A discussion of how structural biology is emerging as a powerful tool for discovering new proteins and interactions, supercharged by advancements in cryo-electron microscopy, cryo-electron tomography and machine learning-based structure prediction.

## Introduction

The forward march of biology is driven by the ability to directly observe living systems at ever-finer levels of detail. As Richard Feynman famously stated: “It is very easy to answer many of these fundamental biological questions; you just look at the thing!” (Feynman, 1960). Light microscopes of the 17th century unveiled a world invisible to the naked eye, with pioneering microscopists like Antonie van Leeuwenhoek describing new microscopic organisms nearly everywhere they looked (Wollman et al., 2015). Three centuries later, the electron microscope pulled back the curtain on the complex inner workings of the cell, spurring rapid discoveries of new subcellular structures with astounding variation across different types of cells and tissues (Knott and Genoud, 2013).

In this Perspective, I argue that the electron microscope is an even more powerful discovery tool in the 21st century, supercharged by advancements in cryo-electron microscopy (cryo-EM) and machine learning-enabled protein structure prediction. Cryo-EM enables imaging of biological systems in a near-native state at the molecular scale (Box 1), and incredible progress in the technique over the past ∼10–15 years has expanded the range of samples amenable to high-resolution structural analysis. Perhaps the best-known upside of cryo-EM single-particle analysis (SPA) (Box 1) is that it obviates the need to purify very large quantities of protein and coax them to crystallize. Indeed, cryo-EM is extremely versatile with regards to input material; through image processing and classification, cryo-EM can solve structures of individual components in heterogenous mixtures such as cell lysates, essentially purifying complexes computationally (Arimura et al., 2021; Han et al., 2025; Ho et al., 2020; Kastritis et al., 2017; Kyrilis et al., 2021a; Lyu et al., 2023; Morgan et al., 2022; Sae-Lee et al., 2022; Schmidt et al., 2024; Skalidis et al., 2022; Su et al., 2021, 2023; Tringides et al., 2023; Tüting et al., 2021; Verbeke et al., 2020). More recently, cryo-focused ion beam (cryo-FIB) milling, cryo-electron tomography (cryo-ET) and subtomogram averaging (Box 1) have made it possible to directly visualize proteins within the native cellular environment at molecular or even near-atomic resolutions (Tegunov et al., 2021; Xue et al., 2022).

Box 1. Cryo-EM sample preparation and imaging modalities Cryo-EM (cryo-electron microscopy or electron cryo-microscopy) encompasses a range of techniques that use electrons to image frozen-hydrated biological material at cryogenic temperatures. Samples (proteins, cells or tissues) are frozen so rapidly that ice does not crystallize and instead forms a glass-like or vitreous state. The samples are imaged directly, without heavy metal staining, so signal comes directly from the biological objects. Thus, cryo-EM combines near-native like structural preservation with high-resolution imaging.

Whereas proteins, cell lysates and smaller cells can be vitrified directly by rapidly plunging them into liquid cryogen (so-called plunge-freezing) (Dubochet and McDowall, 1981), larger cells and tissues require high-pressure freezing to retard ice crystal nucleation and ensure vitrification throughout the sample volume (Dahl and Staehelin, 1989). Thin samples can be imaged directly by cryo-EM, but samples thicker than ∼1 µm are not suitable for imaging (Baumeister, 2002) because more electrons are lost by inelastic scattering and thus mainly contribute noise to the final image. Larger samples must therefore be thinned by cryo-focused ion beam (cryo-FIB) milling (Marko et al., 2007; Rigort et al., 2012) or by physical sectioning (cryo-electron microscopy of vitreous sections; CEMOVIS) (Al-Amoudi et al., 2004).

The main cryo-EM imaging modalities are single-particle analysis (SPA) and cryo-electron tomography (cryo-ET). Both methods aim to generate a faithful three-dimensional (3D) reconstruction from two-dimensional projection images. In SPA, projection images are collected from large numbers of particles, ideally randomly oriented in thin ice (Cheng et al., 2015). The orientations of each particle are determined relative to a reference, and a 3D reconstruction is calculated from the aligned particles. In cryo-ET, projection images are collected from a particular location at different tilt angles, then computationally aligned and back-projected to yield a 3D volume called a tomogram (Turk and Baumeister, 2020). When multiple copies of the same complex are present, subvolumes can be extracted and aligned in a process called subtomogram averaging, enhancing signal-to-noise and increasing resolution (Wan and Briggs, 2016).

Traditionally, cryo-EM SPA is applied to purified proteins, whereas cryo-ET and subtomogram averaging are used for more complex systems like cells or organelles. However, this is not a strict distinction – cryo-ET can be performed on (semi-)purified complexes and SPA can also be performed on vesicles and even organelles, cells or cellular lamellae, provided the particle projections can still be reliably aligned despite the presence of overlapping signal. Indeed, many exciting new developments (some described in this article) blur the lines between the techniques or apply them in concert to leverage their individual strengths.

Cryo-EM can therefore yield structural information about targets that were previously intractable. For instance, macromolecular complexes that cannot be recombinantly expressed, reconstituted *in vitro* or otherwise purified to homogeneity can instead be gently isolated from primary material or imaged directly within intact or partially disrupted cells or organelles. Because protocols with fewer manipulation steps are more likely to preserve fragile protein interactions, a consequence of working with native material is that it becomes increasingly common to encounter unknown proteins, either as unexpected components of a known complex or as completely unidentified structures in raw images or tomograms. Fortunately, it is not necessary to know the identity of a protein complex to reconstruct it in 3D. The main requirements are being able to recognize the complex in raw data, to image many instances of the complex and to align these images computationally. With a cryo-EM map in hand, the task of identifying unknown proteins can be relatively straightforward thanks to an expanding suite of computational approaches, including machine learning-based tools, such as AlphaFold (Jumper et al., 2021).

This leads to a ‘structure-first approach’ in which a cryo-EM map is first calculated, after which molecular composition can be determined directly from the reconstruction. Importantly, this approach requires no prior knowledge of the molecules being reconstructed, and does not necessarily require specific labelling, making cryo-EM of native samples a powerful and versatile approach for protein discovery applicable even to biological systems that have limited molecular or genetic tools available. This Perspective article summarizes general strategies for identifying unknown proteins in cryo-EM maps, then highlights recent examples of how cryo-EM of native samples has led to the discovery of new proteins and interactions in diverse biological contexts.

## Strategies for identifying unknown proteins from cryo-EM density maps

**Fig. 1.**
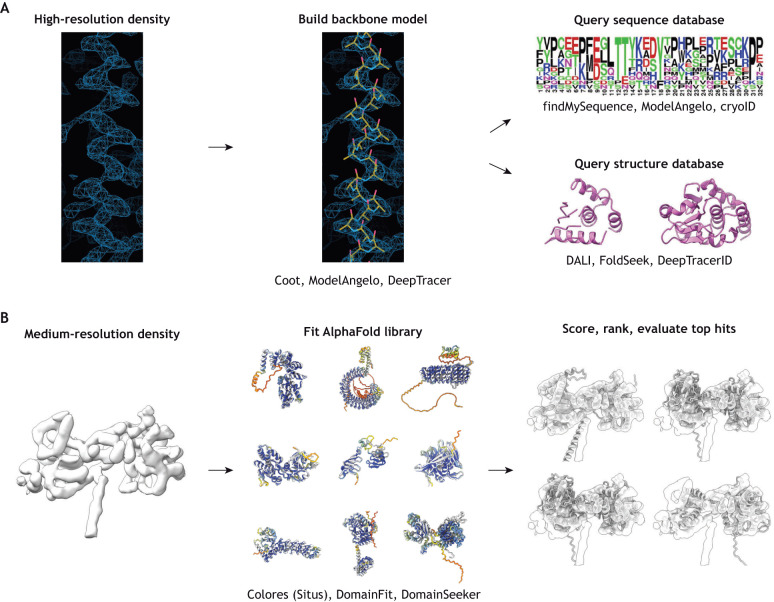
**Workflows for assigning proteins to unknown densities in cryo-EM maps.** (A) For high-resolution regions, a backbone model is built into the map. Side chain densities at each position are assessed and used to query a sequence database to find the most likely candidate. Alternatively, the backbone model can be used to directly query a structure database to find proteins with similar folds. (B) For medium-resolution regions, a library of known or predicted structures can be rigid body-fitted into the map. The fits can be scored and ranked or otherwise assessed to determine the best fit. Images in B are published from Leung et al., 2025, where they were published under CC-BY 4.0 terms. Particular packages used at each stage are noted.

The most efficient strategy for identifying proteins *de novo* from a cryo-EM map depends primarily on what features are visible in the reconstruction (Fig. 1). At low resolutions (>10 Å; 1 Å=0.1 nm), only the overall shape of the protein can be visualized; at intermediate resolution (∼5–7 Å), secondary structure elements can be traced; at high resolutions (below ∼4 Å), amino acid side chains can be resolved, with bulky side chains distinguishable at ∼3–4 Å and smaller side chains distinguishable at ∼3 Å or better.

When side chain densities can be distinguished, it is possible to use a sequence-based approach (Fig. 1A). Here, the protein backbone is traced either manually, using modelling software like Coot (Emsley et al., 2010), or automatically, using machine learning-based tools like DeepTracer (Pfab et al., 2021), CryoNet (Xu et al., 2019) or ModelAngelo (Jamali et al., 2024). Programs like findMySequence (Chojnowski et al., 2022), cryoID (Ho et al., 2020) or ModelAngelo itself can then be used to assess side chain density and identify the most likely candidate from a sequence database. Alternatively, DeepTracer and ModelAngelo also predict the sequences of modelled fragments, which can be used as input for a separate basic local alignment search tool (BLAST) search. Experienced structural biologists might also be able to infer sequence motifs directly from characteristic shapes of side chain densities (Jiang et al., 2022; Khalifa et al., 2020; Schweighauser et al., 2022), but the aforementioned programs make this process more automated and more objective.

In intermediate-resolution maps where secondary structure elements are visible, fold-based approaches have proven effective means to identify proteins (Fig. 1B). In this strategy, programs like DomainFit (Gao et al., 2024), DomainSeeker (Lu et al., 2024 preprint) or the colores tool in the Situs package (Chacón and Wriggers, 2002; Chen et al., 2023a; Wriggers et al., 1999) are used to automatically rigid body fit (i.e. to directly fit without deformation) a library of protein structures into the unknown density, then to score and rank the top hits. Note that fold-based approaches can also be used for high-resolution maps (Fig. 1A); in these cases, the initial backbone trace can be compared to a database of structures through protein comparison servers, such as DALI (Holm, 2022; Holm and Rosenström, 2010), DeepTracer-ID (Chang et al., 2022b) or FoldSeek (van Kempen et al., 2024). Machine learning-based protein structure prediction tools like AlphaFold make these approaches significantly more powerful by providing extensive libraries of high-quality models against which the unknown density can be queried (Jumper et al., 2021; Varadi et al., 2024). Some studies have also successfully identified unknown proteins from cryo-EM maps using the MOLREP-BALBES pipeline, which uses the program MOLREP to fit domains from the BALBES database containing a curated set of unique folds represented in the Protein Data Bank (PDB) (Brown et al., 2015; Ghanim et al., 2021; Ma et al., 2019), or the Omokage search tool, which compares an unknown structure to PDB or Electron Microscopy Data Bank (EMDB) entries based on overall shape similarity (Skalidis et al., 2022; Suzuki et al., 2016).

Although the above approaches can in principle fish out the correct protein from sequence or structure databases derived from the whole proteome of an organism, it is often very useful to have orthogonal proteomics or cross-linking mass spectrometry data for the specific sample used for cryo-EM (Leung et al., 2025; You et al., 2023). This can greatly facilitate protein identification both by decreasing computational requirements through limiting the subset of candidate proteins and hence the size of the database to search against and can help discriminate between multiple candidate proteins in cases of ambiguity. Approaches synergizing gentle biochemical separation with mass spectrometry of discrete fractions have enabled high-resolution analysis of individual protein assemblies from complex cell lysates (Ho et al., 2020; Kastritis et al., 2017). Proteomics and cross-linking mass spectrometry can also help to identify unknown proteins or to model interactions in maps where resolution is insufficient for unambiguous assignment by either fold-based or sequence-based identification alone, which can be the case with structures derived from *in situ* cryo-ET due to the overall lower throughput and technical difficulty of the method (O'Reilly et al., 2020; Xing et al., 2024).

## Recent examples of structure-guided protein discovery

### Microtubule complexes

One of the most bountiful applications of structure-guided protein discovery has been the mapping of highly complex multi-protein networks associated with axonemal microtubules of cilia and flagella in a wide range of species and cell types (Chen et al., 2023a; Doran et al., 2025; Gui et al., 2021, 2022; Ichikawa et al., 2019; Khalifa et al., 2020; Kubo et al., 2023; Leung et al., 2023, 2025; Ma et al., 2019; Stevens et al., 2025; Walton et al., 2023; Xia et al., 2025; Zhao et al., 2025; Zhou et al., 2023; Zhu et al., 2025). Building on pioneering cryo-ET studies in the 2000s that revealed the overall architecture of axonemal microtubules and associated complexes (Nicastro et al., 2006, 2011), these more recent studies produced high-resolution maps that allowed the authors to assign many proteins *de novo* – in some cases over 100 proteins in a single structure (Leung et al., 2025; Walton et al., 2023; Xia et al., 2025).

In most cases, the authors used cryo-EM single-particle analysis (SPA) of axonemes that been gently disrupted to splay or spread apart the microtubules through mild protease digestion, ATP-induced sliding disintegration or surface tension effects from blotting, although Chen et al. (2023a), Tai et al. (2023), and Zhu et al. (2025) achieved their reconstructions using cryo-ET and subtomogram averaging of cryo-FIB milled sperm flagella. Because axonemal microtubules are large, local resolution varied across the cryo-EM maps and necessitated the use of both sequence- and fold-based approaches to build comprehensive atomic models. Regulatory complexes like the T-shaped radial spokes extending from the microtubule surface were resolved at ∼5–8-Å resolution, likely due to flexibility, so their protein composition was elucidated mainly through fold-based approaches. These strategies were successful in part because regulatory complexes consist mainly of proteins with clear globular domains whose shapes can be resolved even at intermediate resolutions. In contrast, regions closest to the microtubule were better resolved – often at <4 Å – which allowed sequence-based identification of microtubule inner proteins (MIPs), which bind to the microtubule lumen, and microtubule-associated proteins (MAPs), which bind to the external microtubule surface. This is fortunate because most MIPs and MAPs lack clear globular domains and would therefore be difficult to identify unambiguously with fold-based approaches. Indeed, more MIPs and MAPs were able to be identified in reconstructions from SPA, which reached <4 Å, compared to those from subtomogram averaging, which reached resolutions between 4.5 and 6.5 Å.

**Fig. 2.**
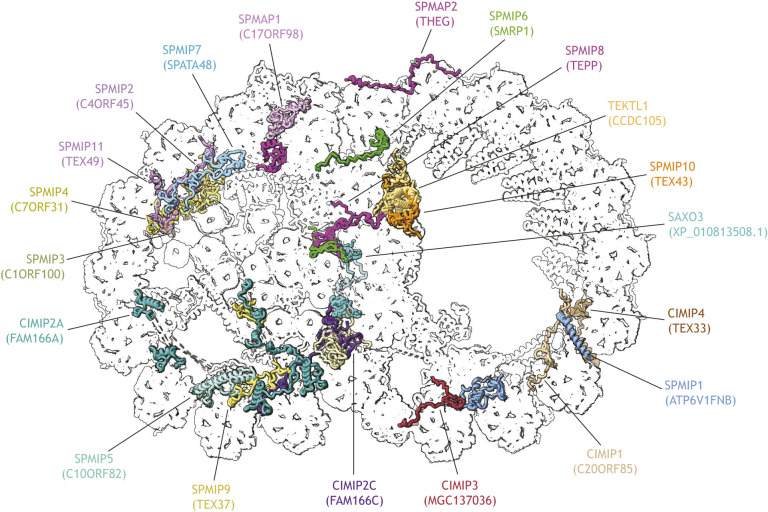
**Previously uncharacterized proteins identified in the cryo-EM map of the 48-nm repeat of native axonemal doublet microtubules from bovine sperm.** The axonemal doublet microtubule structure is from PDB 8OTZ. Uncharacterized proteins with uninformative placeholder names were reclassified as either ciliary microtubule inner proteins (CIMIPs), sperm microtubule inner proteins (SPMIPs), or sperm microtubule associated proteins (SPMAPs).

Together, these structures uncovered the precise binding sites of known proteins implicated in ciliopathies and infertility. More excitingly, they also revealed the identities and interaction networks of previously unknown proteins that represent novel candidate genes associated with male infertility. Among these novel proteins are completely uncharacterized proteins whose uninformative placeholder names (e.g. C#ORF#) reflect the paucity of information about their function or localization. These proteins can now be annotated as microtubule-binding proteins and have thus been renamed ciliary or sperm microtubule inner proteins (CIMIPs or SPMIPs) or microtubule-associated proteins (CIMAPs or SPMAPs), depending on their tissue distribution and their specific localization relative to the microtubule lumen (Leung et al., 2023) (Fig. 2).

A particularly striking finding from the structure of mammalian sperm axonemal microtubules was the discovery of an unexpected binding site for an otherwise well-studied complex not previously thought to associate with the axoneme. Cryo-ET of mammalian sperm flagella initially found that the axonemal radial spokes anchored a prominent barrel-shaped complex, which was not found in the axonemes of any other cell type or species studied (Gadadhar et al., 2021; Leung et al., 2021) and was later shown to be distributed asymmetrically around the axoneme (Chen et al., 2023b). Shortly thereafter, cryo-EM SPA of disintegrated bovine sperm flagella resolved the barrel to ∼7.5 Å, which revealed it to be a fully assembled T-complex protein ring complex (TRiC) chaperone complex owing to the distinct shape of the TRiC complex and of its constituent subunits (Leung et al., 2025). This finding raises questions about whether axoneme-tethered TRiC functions in its canonical role as a chaperone or instead plays a mechanoregulatory role by subtly modifying the flagellar beat.

It is difficult to intuit how such a wealth of information could be derived in any other way as efficiently as through cryo-EM-based *de novo* protein identification. Although several axonemal proteins have been identified in genetically tractable model organisms, such as *Chlamydomonas* or *Tetrahymena*, by integrating cryo-ET with gene disruption or tagging approaches (Dymek et al., 2019; Fu et al., 2018, 2019; Gui et al., 2019; Urbanska et al., 2015), these strategies are difficult to apply to mammalian cilia and sperm flagella. For example, mature sperm are transcriptionally and translationally silent, and the only way to perform genetic perturbations in mature sperm is to generate knockout animals. Furthermore, there is no guarantee that disrupting a single protein causes the corresponding loss of a defined axonemal substructure to which the protein can be mapped.

The structures of axonemal microtubules illustrate how cryo-EM can uncover new proteins binding to known scaffolds, in this case to the microtubule lattice. The success of this field is largely thanks to the fact that axonemal complexes bind in various periodicities that are all in coherent register with the 8-nm repeat of tubulin dimers, the fundamental units of the microtubule. Indeed, cryo-EM has also been used to identify novel microtubule-binding proteins in other contexts, such as in cortical microtubules of the parasite *Toxoplasma* (Wang et al., 2021). To achieve this, the authors treated *Toxoplasma* cells with detergent and directly imaged the resulting cytoskeletons; microtubules could be successfully picked and aligned despite high background noise from cell debris remaining in the sample.

### Filamentous assemblies

Filamentous structures, like microtubules and other cytoskeletal elements, are ubiquitous in biology. Because their striking appearance makes them easy to spot on a cryo-EM grid, it is not entirely uncommon to encounter a filament of unknown composition in a native sample. For example, Cheng et al. (2023, 2024) purified extracellular fibrils from bacterial biofilms using minimal strategies involving only centrifugation and concentration, then solved structures of two unidentified fibrils using cryo-EM SPA. In both cases, the authors used ModelAngelo to automatically build models into high-resolution maps, then identified the proteins through BLAST searches using ModelAngelo-predicted sequences as queries. In a similar vein, Wang et al. (2024a,b, 2025) simply filtered and concentrated pondwater and found diverse filaments with an assortment of shapes and sizes. Without any further fractionation, they used cryo-EM SPA to solve structures of <4 Å for some of these fibrils from an otherwise heterogenous sample. Possibly because of their low abundance and the sheer complexity of the initial sample, these filaments could not be conclusively identified, as no matching sequences were found from proteomics or metagenomics. This challenge highlights an important space for method development that, when addressed, promises to open the door to cryo-EM-guided exploration of environmental samples.

A recent study by Hugener et al. (2024) presented a workflow for identifying unknown filaments that synergizes cryo-ET and subtomogram averaging with cryo-EM SPA. The authors first performed cryo-ET on cryo-FIB-milled lamellae of nutrient-starved yeast cells undergoing gametogenesis and observed uncharacterized filaments in various cellular compartments. They then gently lysed or spread yeast spheroplasts (obtained by treating cells with an enzyme to digest the cell wall) or isolated mitochondria onto cryo-EM grids, which preserved the structure of the filaments while also decreasing sample thickness. This allowed them to collect both cryo-ET and cryo-EM SPA data without the need for tedious FIB milling. The authors used cryo-ET and subtomogram averaging to provide initial low-resolution structures of the filaments that they could then use as initial models to derive helical parameters for subsequent cryo-EM SPA. One type of filament, initially resolved to ∼7 Å, was identified by manually inspecting AlphaFold predictions of mitochondrial proteins that were also upregulated during gametogenesis based on proteomics data; another type of filament, resolved to 3.5 Å, was identified through a DALI search using a manually traced backbone model.

### Membrane proteins

**Fig. 3.**
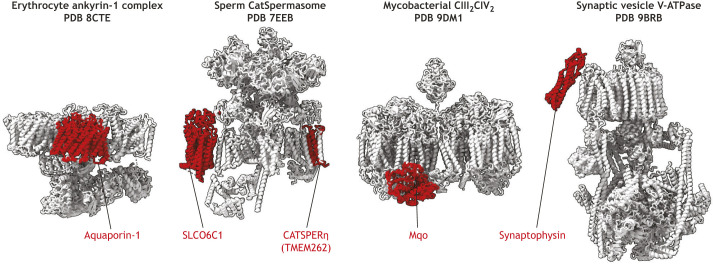
**Selected examples of newly identified components of native membrane protein complexes.** Aquaporin-1 was identified as a component of the ankyrin-1 complex purified from red blood cells (PDB 8CTE). SLCO6C1 and CATSPERη (formerly TMEM262) were described as novel components of the CatSper channel complex (the ‘CatSpermasome’) from mouse sperm (PDB 7EEB). The malate:quinone oxidoreductase Mqo was found to bind the complex III–complex IV supercomplex from mycobacteria (PDB 9DM1). Synaptophysin was identified as a binding partner of V-ATPase from synaptic vesicles (PDB 9BRB).

Cryo-EM has led to the identification of new components of membrane protein complexes, which have traditionally been very challenging structural targets (Fig. 3). Vallese et al. (2022) purified the native ankyrin-1 complex from erythrocyte membranes and solved its structure to <3 Å, which allowed them to unambiguously identify aquaporin as an unexpected component of the complex. In a creative approach combining genetic manipulation with cryo-EM of native samples, Lin et al. (2021) purified the CatSper channel complex, which mediates the influx of Ca^2+^ necessary for sperm hyperactivation, directly from testes and epididymides of transgenic mice expressing a tagged version of one of the known channel subunits. The authors purified the native CatSper complex through affinity purification and found several unexplained densities, which through mass spectrometry they identified as novel auxiliary subunits. This observation could explain why previous attempts to reconstitute the complex were unsuccessful.

Because any form of detergent solubilization risks altering the conformations or interactions of membrane proteins, it would be ideal to solve their structures directly within the bilayer. Recently, several groups have solved structures of membrane proteins natively anchored in cell-derived vesicles and encountered unexplained densities that represented previously unidentified complexes or binding partners that were likely dissociated in previous studies using detergent solubilization. Fu and MacKinnon (2024) prepared native membrane vesicles and observed abundant basket-shaped complexes that they identified as flotillin cages by cryo-EM. Coupland et al. (2024) and Wang et al. (2024a) imaged V-ATPase in native synaptic vesicles using cryo-ET and cryo-EM SPA. Both groups found unexplained densities stoichiometrically associated with the transmembrane regions of V-ATPase, which they identified as synaptophysin by systematically fitting AlphaFold predictions of proteins detected in their preparations. Di Trani et al. (2025) prepared inner membrane vesicles from *Mycobacterium smegmatitis* and affinity-isolated vesicles containing genetically tagged respiratory complexes. The authors found an extra density binding to the complex III–complex IV supercomplex, which they assigned as malate:quinone oxidoreductase (Mqo) by fitting AlphaFold models of known enzymes related to mitochondrial metabolism.

In the studies described above, the use of small vesicles facilitated high-resolution structure determination by allowing the ice on the cryo-EM grids to remain fairly thin, so that high-quality projection images could be collected and analysed by cryo-EM SPA. Another potentially interesting technique for exploring membrane-associated processes is unroofing, in which mechanical shear is applied to cells through, for example, a pressurized fluid. This procedure also washes away most of the cytoplasm, yielding patches of native plasma membrane and associated material that are thin enough to image without cryo-FIB milling (Sun et al., 2025). It even appears to be possible to solve high-resolution structures of membrane proteins from whole organelles by SPA, at least for large complexes such as mitochondria respiratory complexes (Zheng et al., 2024).

There are also encouraging results demonstrating the potential of structure-guided discovery directly in intact cells. In a recent preprint, Jensen et al. (2025) preprint visualized large dome-shaped structures on the plasma membrane of *Mycoplasma pneumoniae* by cellular cryo-ET. They reconstructed the complex at ∼9-Å resolution by subtomogram averaging and, by fitting AlphaFold models of candidates from surface-shaving proteomics (in which intact cells are gently treated with protease to enrich for surface-exposed proteins) and cross-linking mass spectrometry, identified it as a complex of previously uncharacterized proteins that associate with the protein translocation machinery. As a testament to the discovery power of *in situ* cryo-ET, they also found that ribosomes associated with the dome complex, hinting that the complex might function as a chaperone-like folding chamber for newly translated and translocated proteins.

### Pathogenic elements

An intriguing application of the structure-first approach is the identification of disease-related or disease-causing agents in a form of ‘molecular pathology’. For instance, cryo-EM has been used to identify amyloid fibrils isolated from postmortem human brain as being composed of the unexpected protein TMEM106B (Chang et al., 2022a; Jiang et al., 2022; Schweighauser et al., 2022). Because the cryo-EM maps were resolved at <3 Å, protein identity could be deduced by sequence. Interestingly, the three groups used slightly different approaches but converged on the same solution: Schweighauser et al. used sequence motifs deduced from the map to scan the human proteome; Jiang et al. manually inferred the best-fitting amino acid at each position and used the resulting sequence in a BLAST search; and Chang et al. used automated approaches implementing findMySequence and cryoID.

Cryo-EM was also recently used to identify a virus infecting farmed superworm (*Zophobas morio*) larvae (Penzes et al., 2024). The authors isolated viruses directly from carcasses of infected larvae and solved cryo-EM structures to <3 Å. Two independent approaches were used to identify the virus. First, models were built either manually or automatically with ModelAngelo and used as inputs to the DALI server to query the PDB. Second, ModelAngelo-predicted sequences were used to query UniProt. Both approaches yielded proteins from the same viral subfamily as top hits, which informed the choice of an appropriate strategy to sequence the whole viral genome. Impressively, information about viral identity was derived from the cryo-EM map within 1 week of receiving the first samples, illustrating that cryo-EM can provide rapid identification times for ideal samples like abundant, highly symmetric viruses.

## Challenges and prospects

Cryo-EM and cryo-ET are clearly powerful tools, but there are some important considerations that limit the applicability of the structure-first approach. These considerations are inherent to averaging-based approaches: namely, that the target needs to be at least partially structured or repetitive (i.e. ‘averageable’), and the target needs to be abundant in the sample, because large datasets (typically hundreds of thousands to millions of initial particle images) are often needed to reach relatively high resolutions required for protein identification (<∼10 Å), with better resolutions permitting faster and more confident assignments. In practice, this approach is also currently best-suited to protein complexes that are sufficiently large for their particle images to be recognized and aligned despite the potentially high background noise in partially purified or *in situ* samples. Fortunately, it is precisely these large supramolecular assemblies that benefit the most from such an approach as they have complex multi-protein compositions that might not be fully elucidated and are otherwise challenging to study given that they can be difficult to reconstitute or purify to homogeneity without losing components. In the near future, these hurdles will become easier to overcome thanks to advances like improved data collection throughput (Cheng et al., 2018; Eisenstein et al., 2023), better microscope hardware (Nakane et al., 2020; Yip et al., 2020) and image processing pipelines (Kimanius et al., 2024; Tegunov et al., 2021), easier access to high-end instrumentation through shared facilities and national centres (Saibil et al., 2015; Zimanyi et al., 2022), and availability of lower-cost 100-kV electron microscopes (Karia et al., 2025; McMullan et al., 2023).

Many of the examples above are from protein complexes that have been at least partially liberated from the cellular context. However, there are examples from completely intact cellular specimens (Chen et al., 2023a; Jensen et al., 2025) and there are certain to be more such examples enabled by maturation of the technologies and workflows for cryo-FIB milling, cryo-ET and subtomogram averaging. Hybrid workflows using cryo-EM SPA on FIB-milled lamellae also show great promise, with the potential to increase data collection throughput on cellular samples (You et al., 2023; Zheng et al., 2025).

Most structure-based protein discovery projects will likely involve integrating *in situ* cryo-ET and subtomogram averaging with cryo-EM SPA. The initial observation of an unidentified molecular species in cellular cryo-ET data can spur follow-up efforts aimed at reducing the complexity of the system to make targets amenable to cryo-EM SPA while retaining their structures as close as possible to the native state. For abundant targets that are easy to recognize for imaging, gentle disruption through mechanical lysis, hypotonic rupture or mild detergent treatment might suffice. For smaller or rarer targets, it might be necessary to include an enrichment step involving concentration, differential centrifugation, sucrose gradient fractionation or size exclusion chromatography. Once data is collected, even low-resolution maps derived from *in situ* cryo-ET can be used as initial references for alignment. The resulting high-resolution *ex situ* maps can also be compared to these low-resolution cellular reconstructions to determine whether any proteins have been lost during purification.

‘Visual proteomics’ strategies, where fractionated cell lysates are analysed by cryo-EM and mass spectrometry, also represent powerful approaches for systematically exploring the molecular composition of cells (reviewed in Klykov et al., 2022; Kyrilis et al., 2019, 2021b; McCafferty et al., 2020; Ziegler et al., 2021). Excitingly, applying this approach to the *Tetrahymena* ciliary matrix has recently led to the identification and structural characterization of a novel type of protein assembly called the ‘CAGE complex’ that appears to be conserved across broad swaths of the tree of life, encouraging future studies into the functional role of the complex (McCafferty et al., 2025). These studies can likewise complement *in situ* cryo-ET by providing reference data to interpret highly complex cellular tomograms.

It is important to note that cellular cryo-ET and subtomogram averaging are currently the only methods that can report on the true structures of protein complexes *in situ*, and on the subcellular distribution of protein complexes at molecular resolution. For example, recent *in situ* cryo-ET studies have shown that the CatSper channel forms extensive zigzag arrays on the plasma membrane of the mammalian sperm flagellum (Zhao et al., 2022) and that TRiC chaperonin particles in the closed conformation form linear arrays in the cytoplasm (Xing et al., 2024). This information is lost by even the gentlest isolation and purification procedures, but precise knowledge about the spatial arrangement of molecular machines and their conformational states is, in and of itself, an important driver for generating new biological models and hypotheses.

## Concluding remarks

The ability of cryo-EM to produce high-resolution structures from native material is leading to the discovery of novel proteins and interactions in diverse systems. This represents an exciting shift in how structural biology contributes to our broader understanding of biological mechanisms – rather than being the final piece of the puzzle that explains prior biochemical data, a cryo-EM map is now often a starting point for hypothesis generation. Because cryo-EM maps simultaneously contain information about protein identity, structure and interactions, they are important resources for rationally designing downstream functional or genetic studies. Thus, cryo-EM-guided protein discovery could prove to be one of the more prominent roles for experimental structural biology in the post-AlphaFold era. Democratizing access to cryo-EM instrumentation and expertise to accommodate a wider range of projects will broaden the application of this structure-first approach from serendipitous discovery to structured exploration.

## References

[JCS264215C1] Al-Amoudi, A., Chang, J.-J., Leforestier, A., McDowall, A., Salamin, L. M., Norlén, L. P. O., Richter, K., Blanc, N. S., Studer, D. and Dubochet, J. (2004). Cryo-electron microscopy of vitreous sections. *EMBO J.* 23, 3583-3588. 10.1038/sj.emboj.7600366 15318169 PMC517607

[JCS264215C2] Arimura, Y., Shih, R. M., Froom, R. and Funabiki, H. (2021). Structural features of nucleosomes in interphase and metaphase chromosomes. *Mol. Cell* 81, 4377-4397.e12. 10.1016/j.molcel.2021.08.010 34478647 PMC8571072

[JCS264215C3] Baumeister, W. (2002). Electron tomography: Towards visualizing the molecular organization of the cytoplasm. *Curr. Opin. Struct. Biol.* 12, 679-684. 10.1016/S0959-440X(02)00378-0 12464323

[JCS264215C4] Brown, A., Long, F., Nicholls, R. A., Toots, J., Emsley, P. and Murshudov, G. (2015). Tools for macromolecular model building and refinement into electron cryo-microscopy reconstructions. *Acta Crystallogr. D Biol. Crystallogr.* 71, 136-153. 10.1107/S1399004714021683 25615868 PMC4304694

[JCS264215C5] Chacón, P. and Wriggers, W. (2002). Multi-resolution contour-based fitting of macromolecular structures. *J. Mol. Biol.* 317, 375-384. 10.1006/jmbi.2002.5438 11922671

[JCS264215C6] Chang, A., Xiang, X., Wang, J., Lee, C., Arakhamia, T., Simjanoska, M., Wang, C., Carlomagno, Y., Zhang, G., Dhingra, S. et al. (2022a). Homotypic fibrillization of TMEM106B across diverse neurodegenerative diseases. *Cell* 185, 1346-1355.e15. 10.1016/j.cell.2022.02.026 35247328 PMC9018563

[JCS264215C7] Chang, L., Wang, F., Connolly, K., Meng, H., Su, Z., Cvirkaite-Krupovic, V., Krupovic, M., Egelman, E. H. and Si, D. (2022b). DeepTracer-ID: De novo protein identification from cryo-EM maps. *Biophys. J.* 121, 2840-2848. 10.1016/j.bpj.2022.06.025 35769006 PMC9388381

[JCS264215C8] Chen, Z., Shiozaki, M., Haas, K. M., Skinner, W. M., Zhao, S., Guo, C., Polacco, B. J., Yu, Z., Krogan, N. J., Lishko, P. V. et al. (2023a). De novo protein identification in mammalian sperm using in situ cryoelectron tomography and AlphaFold2 docking. *Cell* 186, 5041-5053.e19. 10.1016/j.cell.2023.09.017 37865089 PMC10842264

[JCS264215C9] Chen, Z., Greenan, G. A., Shiozaki, M., Liu, Y., Skinner, W. M., Zhao, X., Zhao, S., Yan, R., Yu, Z., Lishko, P. V. et al. (2023b). In situ cryo-electron tomography reveals the asymmetric architecture of mammalian sperm axonemes. *Nat. Struct. Mol. Biol.* 30, 360-369. 10.1038/s41594-022-00861-0 36593309 PMC10023559

[JCS264215C10] Cheng, Y., Grigorieff, N., Penczek, P. A. and Walz, T. (2015). A primer to single-particle cryo-electron microscopy. *Cell* 161, 438-449. 10.1016/j.cell.2015.03.050 25910204 PMC4409659

[JCS264215C11] Cheng, A., Eng, E. T., Alink, L., Rice, W. J., Jordan, K. D., Kim, L. Y., Potter, C. S. and Carragher, B. (2018). High resolution single particle cryo-electron microscopy using beam-image shift. *J. Struct. Biol.* 204, 270-275. 10.1016/j.jsb.2018.07.015 30055234 PMC6163078

[JCS264215C12] Cheng, Y., Han, J., Song, M., Zhang, S. and Cao, Q. (2023). Serine peptidase Vpr forms enzymatically active fibrils outside Bacillus bacteria revealed by cryo-EM. *Nat. Commun.* 14, 7503. 10.1038/s41467-023-43359-z 37980359 PMC10657474

[JCS264215C13] Cheng, Y., Kreutzberger, M. A. B., Han, J., Egelman, E. H. and Cao, Q. (2024). Molecular architecture of the assembly of Bacillus spore coat protein GerQ revealed by cryo-EM. *Nat. Commun.* 15, 8091. 10.1038/s41467-024-52422-2 39284816 PMC11405398

[JCS264215C14] Chojnowski, G., Simpkin, A. J., Leonardo, D. A., Seifert-Davila, W., Vivas-Ruiz, D. E., Keegan, R. M. and Rigden, D. J. (2022). FindMySequence: a neural-network-based approach for identification of unknown proteins in X-ray crystallography and cryo-EM. *IUCrJ* 9, 86-97. 10.1107/S2052252521011088 PMC8733886 35059213

[JCS264215C15] Coupland, C. E., Karimi, R., Bueler, S. A., Liang, Y., Courbon, G. M., Di Trani, J. M., Wong, C. J., Saghian, R., Youn, J.-Y., Wang, L.-Y. et al. (2024). High-resolution electron cryomicroscopy of V-ATPase in native synaptic vesicles. *Science* 385, 168-174. 10.1126/science.adp5577 38900912

[JCS264215C16] Dahl, R. and Staehelin, L. A. (1989). High-pressure freezing for the preservation of biological structure: Theory and practice. *J. Electron Microsc. Tech.* 13, 165-174. 10.1002/jemt.1060130305 2685196

[JCS264215C17] Di Trani, J. M., Yu, J., Courbon, G. M., Lobez Rodriguez, A. P., Cheung, C.-Y., Liang, Y., Coupland, C. E., Bueler, S. A., Cook, G. M., Brzezinski, P. et al. (2025). Cryo-EM of native membranes reveals an intimate connection between the Krebs cycle and aerobic respiration in mycobacteria. *Proc. Natl Acad. Sci. USA* 122, 2017. 10.1073/pnas.2423761122 PMC11874196 39969994

[JCS264215C18] Doran, M. H., Niu, Q., Zeng, J., Beneke, T., Smith, J., Ren, P., Fochler, S., Coscia, A., Höög, J. L., Meleppattu, S. et al. (2025). Evolutionary adaptations of doublet microtubules in trypanosomatid parasites. *Science* 387, eadr5507. 10.1126/science.adr5507 40080577 PMC7617938

[JCS264215C19] Dubochet, J. and McDowall, A. W. (1981). Vitrification of pure water for electron microscopy. *J. Microsc.* 124, 3-4. 10.1111/j.1365-2818.1981.tb02483.x

[JCS264215C20] Dymek, E. E., Lin, J., Fu, G., Porter, M. E., Nicastro, D. and Smith, E. F. (2019). PACRG and FAP20 form the inner junction of axonemal doublet microtubules and regulate ciliary motility. *Mol. Biol. Cell* 30, 1805-1816. 10.1091/mbc.E19-01-0063 31116684 PMC6727744

[JCS264215C21] Eisenstein, F., Yanagisawa, H., Kashihara, H., Kikkawa, M., Tsukita, S. and Danev, R. (2023). Parallel cryo electron tomography on in situ lamellae. *Nat. Methods* 20, 131-138. 10.1038/s41592-022-01690-1 36456783

[JCS264215C22] Emsley, P., Lohkamp, B., Scott, W. G. and Cowtan, K. (2010). Features and development of Coot. *Acta Crystallogr. D Biol. Crystallogr.* 66, 486-501. 10.1107/S0907444910007493 20383002 PMC2852313

[JCS264215C23] Feynman, R. (1960). There's plenty of room at the bottom. *Eng. Sci.* 23, 22-36.

[JCS264215C24] Fu, Z. and MacKinnon, R. (2024). Structure of the flotillin complex in a native membrane environment. *Proc. Natl. Acad. Sci. USA* 121, e2409334121. 10.1073/pnas.2409334121 38985763 PMC11260169

[JCS264215C25] Fu, G., Wang, Q., Phan, N., Urbanska, P., Joachimiak, E., Lin, J., Wloga, D. and Nicastro, D. (2018). The I1 dynein-associated tether and tether head complex is a conserved regulator of ciliary motility. *Mol. Biol. Cell* 29, 1048-1059. 10.1091/mbc.E18-02-0142 29514928 PMC5921572

[JCS264215C26] Fu, G., Zhao, L., Dymek, E., Hou, Y., Song, K., Phan, N., Shang, Z., Smith, E. F., Witman, G. B. and Nicastro, D. (2019). Structural organization of the C1a-e-c supercomplex within the ciliary central apparatus. *J. Cell Biol.* 218, 4236-4251. 10.1083/jcb.201906006 31672705 PMC6891083

[JCS264215C27] Gadadhar, S., Alvarez Viar, G., Hansen, J. N., Gong, A., Kostarev, A., Ialy-Radio, C., Leboucher, S., Whitfield, M., Ziyyat, A., Touré, A. et al. (2021). Tubulin glycylation controls axonemal dynein activity, flagellar beat, and male fertility. *Science* 371, eabd4914. 10.1126/science.abd4914 33414192 PMC7612590

[JCS264215C28] Gao, J., Tong, M., Lee, C., Gaertig, J., Legal, T. and Bui, K. H. (2024). DomainFit: Identification of protein domains in cryo-EM maps at intermediate resolution using AlphaFold2-predicted models. *Structure* 32, 1248-1259.e5. 10.1016/j.str.2024.04.017 38754431 PMC11316655

[JCS264215C29] Ghanim, G. E., Fountain, A. J., van Roon, A. M. M., Rangan, R., Das, R., Collins, K. and Nguyen, T. H. D. (2021). Structure of human telomerase holoenzyme with bound telomeric DNA. *Nature* 593, 449-453. 10.1038/s41586-021-03415-4 33883742 PMC7610991

[JCS264215C30] Gui, L., Song, K., Tritschler, D., Bower, R., Yan, S., Dai, A., Augspurger, K., Sakizadeh, J., Grzemska, M., Ni, T. et al. (2019). Scaffold subunits support associated subunit assembly in the Chlamydomonas ciliary nexin-dynein regulatory complex. *Proc. Natl. Acad. Sci. USA* 116, 23152-23162. 10.1073/pnas.1910960116 31659045 PMC6859327

[JCS264215C31] Gui, M., Farley, H., Anujan, P., Anderson, J. R., Maxwell, D. W., Whitchurch, J. B., Botsch, J. J., Qiu, T., Meleppattu, S., Singh, S. K. et al. (2021). De novo identification of mammalian ciliary motility proteins using cryo-EM. *Cell* 184, 5791-5806.e19. 10.1016/j.cell.2021.10.007 34715025 PMC8595878

[JCS264215C32] Gui, M., Wang, X., Dutcher, S. K., Brown, A. and Zhang, R. (2022). Ciliary central apparatus structure reveals mechanisms of microtubule patterning. *Nat. Struct. Mol. Biol.* 29, 483-492. 10.1038/s41594-022-00770-2 35578023 PMC9930914

[JCS264215C33] Han, X., Zhang, Z., Su, C. C., Lyu, M., Miyagi, M., Yu, E. and Nieman, M. T. (2025). Elucidating the dynamics of integrin αIIbβ3 from native platelet membranes by cryo-EM with build-and-retrieve method. *Blood Adv.* 9, 4592-4606. 10.1182/bloodadvances.2025016209 40472320 PMC12455098

[JCS264215C34] Ho, C. M., Li, X., Lai, M., Terwilliger, T. C., Beck, J. R., Wohlschlegel, J., Goldberg, D. E., Fitzpatrick, A. W. P. and Zhou, Z. H. (2020). Bottom-up structural proteomics: cryoEM of protein complexes enriched from the cellular milieu. *Nat. Methods* 17, 79-85. 10.1038/s41592-019-0637-y 31768063 PMC7494424

[JCS264215C35] Holm, L. (2022). Dali server: structural unification of protein families. *Nucleic Acids Res.* 50, W210-W215. 10.1093/nar/gkac387 35610055 PMC9252788

[JCS264215C36] Holm, L. and Rosenström, P. (2010). Dali server: Conservation mapping in 3D. *Nucleic Acids Res.* 38, 545-549. 10.1093/nar/gkq366 PMC2896194 20457744

[JCS264215C37] Hugener, J., Xu, J., Wettstein, R., Ioannidi, L., Velikov, D., Wollweber, F., Henggeler, A., Matos, J. and Pilhofer, M. (2024). FilamentID reveals the composition and function of metabolic enzyme polymers during gametogenesis. *Cell* 187, 3303-3318.e18. 10.1016/j.cell.2024.04.026 38906101

[JCS264215C38] Ichikawa, M., Khalifa, A. A. Z., Kubo, S., Dai, D., Basu, K., Maghrebi, M. A. F., Vargas, J. and Bui, K. H. (2019). Tubulin lattice in cilia is in a stressed form regulated by microtubule inner proteins. *Proc. Natl Acad. Sci. USA* 116, 19930-19938. 10.1073/pnas.1911119116 31527277 PMC6778249

[JCS264215C39] Jamali, K., Käll, L., Zhang, R., Brown, A., Kimanius, D. and Scheres, S. H. W. (2024). Automated model building and protein identification in cryo-EM maps. *Nature* 628, 450-457. 10.1038/s41586-024-07215-4 38408488 PMC11006616

[JCS264215C40] Jensen, R. K., Xue, L., Marotta, F., Somody, J. C., Selkrig, J., Lenz, S., Rappsilber, J., Savitski, M. M., Kosinski, J., Typas, A. et al. (2025). In-cell discovery and characterization of a non-canonical bacterial protein translocation-folding complex. *bioRxiv*, 2025.04.25.650208. 10.1101/2025.04.25.650208

[JCS264215C41] Jiang, Y. X., Cao, Q., Sawaya, M. R., Abskharon, R., Ge, P., DeTure, M., Dickson, D. W., Fu, J. Y., Ogorzalek Loo, R. R., Loo, J. A. et al. (2022). Amyloid fibrils in FTLD-TDP are composed of TMEM106B and not TDP-43. *Nature* 605, 304-309. 10.1038/s41586-022-04670-9 35344984 PMC9844993

[JCS264215C42] Jumper, J., Evans, R., Pritzel, A., Green, T., Figurnov, M., Ronneberger, O., Tunyasuvunakool, K., Bates, R., Žídek, A., Potapenko, A. et al. (2021). Highly accurate protein structure prediction with AlphaFold. *Nature* 596, 583-589. 10.1038/s41586-021-03819-2 34265844 PMC8371605

[JCS264215C43] Karia, D., Koh, A. F., Yang, W., Cushing, V. I., Basanta, B., Mihaylov, D. B., Khavnekar, S., Vyroubal, O., Malínský, M., Sháněl, O. et al. (2025). Sub-3 Å resolution protein structure determination by single-particle cryo-EM at 100 keV. *Structure* 33, 1717-1727.e4. 10.1016/j.str.2025.07.007 40744008 PMC12372669

[JCS264215C44] Kastritis, P. L., O'Reilly, F. J., Bock, T., Li, Y., Rogon, M. Z., Buczak, K., Romanov, N., Betts, M. J., Bui, K. H., Hagen, W. J. et al. (2017). Capturing protein communities by structural proteomics in a thermophilic eukaryote. *Mol. Syst. Biol.* 13, 936. 10.15252/msb.20167412 28743795 PMC5527848

[JCS264215C45] Khalifa, A., Ichikawa, M., Dai, D., Kubo, S., Black, C., Peri, K., McAlear, T. S., Veyron, S., Yang, S. K., Vargas, J. et al. (2020). The inner junction complex of the cilia is an interaction hub that involves tubulin post-translational modifications. *eLife* 9, 1-25. 10.7554/eLife.52760 PMC6994238 31951202

[JCS264215C46] Kimanius, D., Jamali, K., Wilkinson, M. E., Lövestam, S., Velazhahan, V., Nakane, T. and Scheres, S. H. W. (2024). Data-driven regularization lowers the size barrier of cryo-EM structure determination. *Nat. Methods* 21, 1216-1221. 10.1038/s41592-024-02304-8 38862790 PMC11239489

[JCS264215C47] Klykov, O., Kopylov, M., Carragher, B., Heck, A. J. R., Noble, A. J. and Scheltema, R. A. (2022). Label-free visual proteomics: Coupling MS- and EM-based approaches in structural biology. *Mol. Cell* 82, 285-303. 10.1016/j.molcel.2021.12.027 35063097 PMC8842845

[JCS264215C48] Knott, G. and Genoud, C. (2013). Is EM dead? *J. Cell Sci.* 126, 4545-4552. 10.1242/jcs.124123 24124192

[JCS264215C49] Kubo, S., Black, C. S., Joachimiak, E., Yang, S. K., Legal, T., Peri, K., Khalifa, A. A. Z., Ghanaeian, A., McCafferty, C. L., Valente-Paterno, M. et al. (2023). Native doublet microtubules from Tetrahymena thermophila reveal the importance of outer junction proteins. *Nat. Commun.* 14, 2168. 10.1038/s41467-023-37868-0 37061538 PMC10105768

[JCS264215C50] Kyrilis, F. L., Meister, A. and Kastritis, P. L. (2019). Integrative biology of native cell extracts: a new era for structural characterization of life processes. *Biol. Chem.* 400, 831-846. 10.1515/hsz-2018-0445 31091193

[JCS264215C51] Kyrilis, F. L., Semchonok, D. A., Skalidis, I., Tüting, C., Hamdi, F., O'Reilly, F. J., Rappsilber, J. and Kastritis, P. L. (2021a). Integrative structure of a 10-megadalton eukaryotic pyruvate dehydrogenase complex from native cell extracts. *Cell Rep.* 34, 108727. 10.1016/j.celrep.2021.108727 33567276

[JCS264215C52] Kyrilis, F. L., Belapure, J. and Kastritis, P. L. (2021b). Detecting protein communities in native cell extracts by machine learning: a structural biologist's perspective. *Front. Mol. Biosci.* 8, 660542. 10.3389/fmolb.2021.660542 33937337 PMC8082361

[JCS264215C53] Leung, M. R., Roelofs, M. C., Ravi, R. T., Maitan, P., Henning, H., Zhang, M., Bromfield, E. G., Howes, S. C., Gadella, B. M., Bloomfield-Gadêlha, H. et al. (2021). The multi-scale architecture of mammalian sperm flagella and implications for ciliary motility. *EMBO J.* 40, e107410. 10.15252/embj.2020107410 33694216 PMC8013824

[JCS264215C54] Leung, M. R., Zeng, J., Wang, X., Roelofs, M. C., Huang, W., Zenezini Chiozzi, R., Hevler, J. F., Heck, A. J. R., Dutcher, S. K., Brown, A. et al. (2023). Structural specializations of the sperm tail. *Cell* 186, 2880-2896.e17. 10.1016/j.cell.2023.05.026 37327785 PMC10948200

[JCS264215C55] Leung, M. R., Sun, C., Zeng, J., Anderson, J. R., Niu, Q., Huang, W., Noteborn, W. E. M., Brown, A., Zeev-Ben-Mordehai, T. and Zhang, R. (2025). Structural diversity of axonemes across mammalian motile cilia. *Nature* 637, 1170-1177. 10.1038/s41586-024-08337-5 39743588 PMC11779644

[JCS264215C56] Lin, S., Ke, M., Zhang, Y., Yan, Z. and Wu, J. (2021). Structure of a mammalian sperm cation channel complex. *Nature* 595, 746-750. 10.1038/s41586-021-03742-6 34225353

[JCS264215C57] Lu, Y., Chen, G., Sun, F., Zhu, Y. and Zhang, Z. (2024). De novo identification of protein domains in cryo-electron tomography maps from AlphaFold2 models. *bioRxiv*, 2024.11.21.623534. 10.1101/2024.11.21.623534

[JCS264215C58] Lyu, M., Su, C. C., Miyagi, M. and Yu, E. W. (2023). Simultaneous solving high-resolution structures of various enzymes from human kidney microsomes. *Life Sci. Alliance* 6, e202201580. 10.26508/lsa.202201580 36450445 PMC9713302

[JCS264215C59] Ma, M., Stoyanova, M., Rademacher, G., Dutcher, S. K., Brown, A. and Zhang, R. (2019). Structure of the decorated ciliary doublet microtubule. *Cell* 179, 909-922.e12. 10.1016/j.cell.2019.09.030 31668805 PMC6936269

[JCS264215C60] Marko, M., Hsieh, C., Schalek, R., Frank, J. and Mannella, C. (2007). Focused-ion-beam thinning of frozen-hydrated biological specimens for cryo-electron microscopy. *Nat. Methods* 4, 215-217. 10.1038/nmeth1014 17277781

[JCS264215C61] McCafferty, C. L., Verbeke, E. J., Marcotte, E. M. and Taylor, D. W. (2020). Structural biology in the multi-omics era. *J. Chem. Inf. Model.* 60, 2424-2429. 10.1021/acs.jcim.9b01164 32129623 PMC7254829

[JCS264215C62] McCafferty, C. L., Hoogerbrugge, G., Papoulas, O., Schwartz, E. A., Ritchey, S., Taylor, D. W., Brilot, A. F. and Marcotte, E. M. (2025). The CAGE complex: a hollow, megadalton, protein assembly in prokaryotic and eukaryotic microbes. *bioRxiv*, 2025.09.22.677704. 10.1101/2025.09.22.677704

[JCS264215C63] McMullan, G., Naydenova, K., Mihaylov, D., Yamashita, K., Peet, M. J., Wilson, H., Dickerson, J. L., Chen, S., Cannone, G., Lee, Y. et al. (2023). Structure determination by cryoEM at 100 keV. *Proc. Natl. Acad. Sci. USA* 120, 1-8. 10.1073/pnas.2312905120 PMC10710074 38011573

[JCS264215C64] Morgan, C. E., Zhang, Z., Miyagi, M., Golczak, M. and Yu, E. W. (2022). Toward structural-omics of the bovine retinal pigment epithelium. *Cell Rep.* 41, 111876. 10.1016/j.celrep.2022.111876 36577381 PMC9875382

[JCS264215C65] Nakane, T., Kotecha, A., Sente, A., McMullan, G., Masiulis, S., Brown, P. M. G. E., Grigoras, I. T., Malinauskaite, L., Malinauskas, T., Miehling, J. et al. (2020). Single-particle cryo-EM at atomic resolution. *Nature* 587, 152-156. 10.1038/s41586-020-2829-0 33087931 PMC7611073

[JCS264215C66] Nicastro, D., Schwartz, C., Pierson, J., Gaudette, R., Porter, M. E. and McIntosh, J. R. (2006). The molecular architecture of axonemes revealed by cryoelectron tomography. *Science* 313, 944-948. 16917055 10.1126/science.1128618

[JCS264215C67] Nicastro, D., Fu, X., Heuser, T., Tso, A., Porter, M. E. and Linck, R. W. (2011). Cryo-electron tomography reveals conserved features of doublet microtubules in flagella. *Proc. Natl Acad. Sci. USA* 108, E845-E853. 10.1073/pnas.1106178108 21930914 PMC3198354

[JCS264215C68] O'Reilly, F. J., Xue, L., Graziadei, A., Sinn, L., Lenz, S., Tegunov, D., Blötz, C., Singh, N., Hagen, W. J. H., Cramer, P. et al. (2020). In-cell architecture of an actively transcribing-translating expressome. *Science* 369, 554-557. 32732422 10.1126/science.abb3758 PMC7115962

[JCS264215C69] Penzes, J. J., Holm, M., Yost, S. A. and Kaelber, J. T. (2024). Cryo-EM-based discovery of a pathogenic parvovirus causing epidemic mortality by black wasting disease in farmed beetles. *Cell* 187, 5604-5619.e14. 10.1016/j.cell.2024.07.053 39208798 PMC11781814

[JCS264215C70] Pfab, J., Phan, N. M. and Si, D. (2021). DeepTracer for fast de novo cryo-EM protein structure modeling and special studies on cov-related complexes. *Proc. Natl. Acad. Sci. USA* 118, e2017525118. 10.1073/pnas.2017525118 33361332 PMC7812826

[JCS264215C71] Rigort, A., Bauerlein, F. J. B., Villa, E., Eibauer, M., Laugks, T., Baumeister, W. and Plitzko, J. M. (2012). Focused ion beam micromachining of eukaryotic cells for cryoelectron tomography. *Proc. Natl Acad. Sci. USA* 109, 4449-4454. 10.1073/pnas.1201333109 22392984 PMC3311327

[JCS264215C72] Sae-Lee, W., McCafferty, C. L., Verbeke, E. J., Havugimana, P. C., Papoulas, O., McWhite, C. D., Houser, J. R., Vanuytsel, K., Murphy, G. J., Drew, K. et al. (2022). The protein organization of a red blood cell. *Cell Rep.* 40, 111103. 10.1016/j.celrep.2022.111103 35858567 PMC9764456

[JCS264215C73] Saibil, H. R., Grünewald, K. and Stuart, D. I. (2015). A national facility for biological cryo-electron microscopy. *Acta Crystallogr. D Biol. Crystallogr.* 71, 127-135. 10.1107/S1399004714025280 25615867 PMC4304693

[JCS264215C74] Schmidt, L., Tüting, C., Kyrilis, F. L., Hamdi, F., Semchonok, D. A., Hause, G., Meister, A., Ihling, C., Stubbs, M. T., Sinz, A. et al. (2024). Delineating organizational principles of the endogenous L-A virus by cryo-EM and computational analysis of native cell extracts. *Commun. Biol.* 7, 557. 10.1038/s42003-024-06204-7 38730276 PMC11087493

[JCS264215C75] Schweighauser, M., Arseni, D., Bacioglu, M., Huang, M., Lövestam, S., Shi, Y., Yang, Y., Zhang, W., Kotecha, A., Garringer, H. J. et al. (2022). Age-dependent formation of TMEM106B amyloid filaments in human brains. *Nature* 605, 310-314. 10.1038/s41586-022-04650-z 35344985 PMC9095482

[JCS264215C76] Skalidis, I., Kyrilis, F. L., Tüting, C., Hamdi, F., Chojnowski, G. and Kastritis, P. L. (2022). Cryo-EM and artificial intelligence visualize endogenous protein community members. *Structure* 30, 575-589.e6. 10.1016/j.str.2022.01.001 35093201

[JCS264215C77] Stevens, A., Kashyap, S., Crofut, E. H., Wang, S. E., Muratore, K. A., Johnson, P. J. and Hong Zhou, Z. (2025). Structures of native doublet microtubules from trichomonas vaginalis reveal parasite-specific proteins. *Nat. Commun.* 16, 3996. 10.1038/s41467-025-59369-y 40301421 PMC12041511

[JCS264215C78] Su, C.-C., Lyu, M., Morgan, C. E., Bolla, J. R., Robinson, C. V. and Yu, E. W. (2021). A ‘Build and Retrieve’ methodology to simultaneously solve cryo-EM structures of membrane proteins. *Nat. Methods* 18, 69-75. 10.1038/s41592-020-01021-2 33408407 PMC7808410

[JCS264215C79] Su, C. C., Lyu, M., Zhang, Z., Miyagi, M., Huang, W., Taylor, D. J. and Yu, E. W. (2023). High-resolution structural-omics of human liver enzymes. *Cell Rep.* 42, 112609. 10.1016/j.celrep.2023.112609 37289586 PMC10592444

[JCS264215C80] Sun, W. W., Michalak, D. J., Sochacki, K. A., Kunamaneni, P., Alfonzo-Méndez, M. A., Arnold, A. M., Strub, M.-P., Hinshaw, J. E. and Taraska, J. W. (2025). Cryo-electron tomography pipeline for plasma membranes. *Nat. Commun.* 16, 855. 10.1038/s41467-025-56045-z 39833141 PMC11747107

[JCS264215C81] Suzuki, H., Kawabata, T. and Nakamura, H. (2016). Omokage search: Shape similarity search service for biomolecular structures in both the PDB and EMDB. *Bioinformatics* 32, 619-620. 10.1093/bioinformatics/btv614 26508754 PMC4743628

[JCS264215C82] Tai, L., Yin, G., Huang, X., Sun, F. and Zhu, Y. (2023). In-cell structural insight into the stability of sperm microtubule doublet. *Cell Discov.* 9, 116. 10.1038/s41421-023-00606-3 37989994 PMC10663601

[JCS264215C83] Tegunov, D., Xue, L., Dienemann, C., Cramer, P. and Mahamid, J. (2021). Multi-particle cryo-EM refinement with M visualizes ribosome-antibiotic complex at 3.5 Å in cells. *Nat. Methods* 18, 186-193. 10.1038/s41592-020-01054-7 33542511 PMC7611018

[JCS264215C84] Tringides, M. L., Zhang, Z., Morgan, C. E., Su, C. C. and Yu, E. W. (2023). A cryo-electron microscopic approach to elucidate protein structures from human brain microsomes. *Life Sci. Alliance* 6, e202201724. 10.26508/lsa.202201724 36450447 PMC9713474

[JCS264215C85] Turk, M. and Baumeister, W. (2020). The promise and the challenges of cryo-electron tomography. *FEBS Lett.* 594, 3243-3261. 10.1002/1873-3468.13948 33020915

[JCS264215C86] Tüting, C., Kyrilis, F. L., Müller, J., Sorokina, M., Skalidis, I., Hamdi, F., Sadian, Y. and Kastritis, P. L. (2021). Cryo-EM snapshots of a native lysate provide structural insights into a metabolon-embedded transacetylase reaction. *Nat. Commun.* 12, 1-13. 10.1038/s41467-021-27287-4 34836937 PMC8626477

[JCS264215C87] Urbanska, P., Song, K., Joachimiak, E., Krzemien-Ojak, L., Koprowski, P., Hennessey, T., Jerka-Dziadosz, M., Fabczak, H., Gaertig, J., Nicastro, D. et al. (2015). The CSC proteins FAP61 and FAP251 build the basal substructures of radial spoke 3 in cilia. *Mol. Biol. Cell* 26, 1463-1475. 10.1091/mbc.E14-11-1545 25694453 PMC4395127

[JCS264215C88] Vallese, F., Kim, K., Yen, L. Y., Johnston, J. D., Noble, A. J., Calì, T. and Clarke, O. B. (2022). Architecture of the human erythrocyte ankyrin-1 complex. *Nat. Struct. Mol. Biol.* 29, 706-718. 10.1038/s41594-022-00792-w 35835865 PMC10373098

[JCS264215C89] van Kempen, M., Kim, S. S., Tumescheit, C., Mirdita, M., Lee, J., Gilchrist, C. L. M., Söding, J. and Steinegger, M. (2024). Fast and accurate protein structure search with Foldseek. *Nat. Biotechnol.* 42, 243-246. 10.1038/s41587-023-01773-0 37156916 PMC10869269

[JCS264215C90] Varadi, M., Bertoni, D., Magana, P., Paramval, U., Pidruchna, I., Radhakrishnan, M., Tsenkov, M., Nair, S., Mirdita, M., Yeo, J. et al. (2024). AlphaFold Protein Structure Database in 2024: providing structure coverage for over 214 million protein sequences. *Nucleic Acids Res.* 52, D368-D375. 10.1093/nar/gkad1011 37933859 PMC10767828

[JCS264215C91] Verbeke, E. J., Zhou, Y., Horton, A. P., Mallam, A. L., Taylor, D. W. and Marcotte, E. M. (2020). Separating distinct structures of multiple macromolecular assemblies from cryo-EM projections. *J. Struct. Biol.* 209, 107416. 10.1016/j.jsb.2019.107416 31726096 PMC6952565

[JCS264215C92] Walton, T., Gui, M., Velkova, S., Fassad, M. R., Hirst, R. A., Haarman, E., O'Callaghan, C., Bottier, M., Burgoyne, T., Mitchison, H. M. et al. (2023). Axonemal structures reveal mechanoregulatory and disease mechanisms. *Nature* 618, 625-633. 10.1038/s41586-023-06140-2 37258679 PMC10266980

[JCS264215C93] Wan, W. and Briggs, J. A. G. (2016). Cryo-electron tomography and subtomogram averaging. *Methods Enzymol.* 579, 329-367. 10.1016/bs.mie.2016.04.014 27572733

[JCS264215C94] Wang, X., Fu, Y., Beatty, W. L., Ma, M., Brown, A., David Sibley, L. and Zhang, R. (2021). Cryo-EM structure of cortical microtubules from human parasite Toxoplasma gondii identifies their microtubule inner proteins. *Nat. Commun.* 12, 3065. 10.1038/s41467-021-23351-1 34031406 PMC8144581

[JCS264215C95] Wang, C., Jiang, W., Leitz, J., Yang, K., Esquivies, L., Wang, X., Shen, X., Held, R. G., Adams, D. J., Basta, T. et al. (2024a). Structure and topography of the synaptic V-ATPase–synaptophysin complex. *Nature* 631, 899-904. 10.1038/s41586-024-07610-x 38838737 PMC11269182

[JCS264215C96] Wang, T., Li, Z., Xu, K., Huang, W., Huang, G., Zhang, Q. C. and Yan, N. (2024b). CryoSeek: a strategy for bioentity discovery using cryoelectron microscopy. *Proc. Natl. Acad. Sci. USA* 121, e2417046121. 10.1073/pnas.2417046121 39382995 PMC11494351

[JCS264215C97] Wang, T., Huang, W., Xu, K., Sun, Y., Zhang, Q. C., Yan, C., Li, Z. and Yan, N. (2025). CryoSeek II: Cryo-EM analysis of glycofibrils from freshwater reveals well-structured glycans coating linear tetrapeptide repeats. *Proc. Natl Acad. Sci. USA* 122, e2423943122. 10.1073/pnas.2423943122 39739783 PMC11725842

[JCS264215C98] Wollman, A. J. M., Nudd, R., Hedlund, E. G. and Leake, M. C. (2015). From Animaculum to single molecules: 300 years of the light microscope. *Open Biol.* 5, 150019. 10.1098/rsob.150019 25924631 PMC4422127

[JCS264215C99] Wriggers, W., Milligan, R. A. and McCammon, J. A. (1999). Situs: a package for docking crystal structures into low-resolution maps from electron microscopy. *J. Struct. Biol.* 125, 185-195. 10.1006/jsbi.1998.4080 10222274

[JCS264215C100] Xia, X., Shimogawa, M. M., Wang, H., Liu, S., Wijono, A., Langousis, G., Kassem, A. M., Wohlschlegel, J. A., Hill, K. L. and Zhou, Z. H. (2025). Trypanosome doublet microtubule structures reveal flagellum assembly and motility mechanisms. *Science* 387, eadr3314. 10.1126/science.adr3314 40080582 PMC12165780

[JCS264215C101] Xing, H., Rosenkranz, R. R. E., Rodriguez-Aliaga, P., Lee, T. T., Majtner, T., Böhm, S., Turoňová, B., Frydman, J. and Beck, M. (2024). In situ analysis reveals the TRiC duty cycle and PDCD5 as an open-state cofactor. *Nature* 637, 983-990. 10.1038/s41586-024-08321-z 39663456 PMC11754096

[JCS264215C102] Xu, K., Wang, Z., Shi, J., Li, H. and Zhang, Q. C. (2019). A2-Net: Molecular Structure Estimation from Cryo-EM Density Volumes. Proceedings of the AAAI Conference on Artificial Intelligence, 1230-1237. 10.1609/aaai.v33i01.33011230

[JCS264215C103] Xue, L., Lenz, S., Zimmermann-Kogadeeva, M., Tegunov, D., Cramer, P., Bork, P., Rappsilber, J. and Mahamid, J. (2022). Visualizing translation dynamics at atomic detail inside a bacterial cell. *Nature* 610, 205-211. 10.1038/s41586-022-05255-2 36171285 PMC9534751

[JCS264215C104] Yip, K. M., Fischer, N., Paknia, E., Chari, A. and Stark, H. (2020). Atomic-resolution protein structure determination by cryo-EM. *Nature* 587, 157-161. 10.1038/s41586-020-2833-4 33087927

[JCS264215C105] You, X., Zhang, X., Cheng, J., Xiao, Y., Ma, J., Sun, S., Zhang, X., Wang, H. W. and Sui, S. F. (2023). In situ structure of the red algal phycobilisome–PSII–PSI–LHC megacomplex. *Nature* 616, 199-206. 10.1038/s41586-023-05831-0 36922595

[JCS264215C106] Zhao, Y., Wang, H., Wiesehoefer, C., Shah, N. B., Reetz, E., Hwang, J. Y., Huang, X., Wang, T., Lishko, P. V., Davies, K. M. et al. (2022). 3D structure and in situ arrangements of CatSper channel in the sperm flagellum. *Nat. Commun.* 13, 3439. 10.1038/s41467-022-31050-8 35715406 PMC9205950

[JCS264215C107] Zhao, Y., Song, K., Tavakoli, A., Gui, L., Fernandez-Gonzalez, A., Zhang, S., Dzeja, P. P., Mitsialis, S. A., Zhang, X. and Nicastro, D. (2025). Mouse radial spoke 3 is a metabolic and regulatory hub in cilia. *Nat. Struct. Mol. Biol.* 32, 1542-1554. 10.1038/s41594-025-01594-6 40579595 PMC12451722

[JCS264215C108] Zheng, W., Chai, P., Zhu, J. and Zhang, K. (2024a). High-resolution in situ structures of mammalian respiratory supercomplexes. *Nature* 631, 232-239. 10.1038/s41586-024-07488-9 38811722 PMC11222160

[JCS264215C109] Zheng, W., Zhang, Y., Wang, J., Wang, S., Chai, P., Bailey, E. J., Zhu, C., Guo, W., Devarkar, S. C., Wu, S. et al. (2025). Visualizing the translation landscape in human cells at high resolution. *Nat. Commun.* 16, 10757. 10.1038/s41467-025-65795-9 41315256 PMC12663405

[JCS264215C110] Zhou, L., Liu, H., Liu, S., Yang, X., Dong, Y., Pan, Y., Xiao, Z., Zheng, B., Sun, Y., Huang, P. et al. (2023). Structures of sperm flagellar doublet microtubules expand the genetic spectrum of male infertility. *Cell* 186, 2897-2910.e19. 10.1016/j.cell.2023.05.009 37295417

[JCS264215C111] Zhu, Y., Lin, T., Yin, G., Tai, L., Chen, L., Ma, J., Huang, G., Lu, Y., Zhang, Z., Wang, B. et al. (2025). In situ structure of the mouse sperm central apparatus reveals mechanistic insights into asthenozoospermia. *Cell Res.* 35, 551-567. 10.1038/s41422-025-01135-2 40473901 PMC12297659

[JCS264215C112] Ziegler, S. J., Mallinson, S. J. B., St. John, P. C. and Bomble, Y. J. (2021). Advances in integrative structural biology: towards understanding protein complexes in their cellular context. *Comput. Struct. Biotechnol. J.* 19, 214-225. 10.1016/j.csbj.2020.11.052 33425253 PMC7772369

[JCS264215C113] Zimanyi, C. M., Kopylov, M., Potter, C. S., Carragher, B. and Eng, E. T. (2022). Broadening access to cryoEM through centralized facilities. *Trends Biochem. Sci.* 47, 106-116. 10.1016/j.tibs.2021.10.007 34823974 PMC8760164

